# Letter to the Editor: Non-selective bilateral internal iliac artery embolization is a safe and effective way in hemorrhage control for hemodynamically unstable pelvic fractures

**DOI:** 10.1186/s12891-021-04212-w

**Published:** 2021-04-14

**Authors:** Hui Li, Ping Hu

**Affiliations:** Department of Traumatology, Chongqing University Central Hospital, Chongqing Emergency Medical Center, 1 Jiankang Road, 400014, Yuzhong District, Chongqing, China

**Keywords:** Pelvic fracture, Infection, Angiography, Embolization, Internal iliac artery

## Abstract

A recently published article by Lai et al. in BMC Musculoskeletal Disorders trying to show that patients with pelvic fractures undergoing non-selective internal iliac artery embolization may lead to a higher rate of surgical site infection. The authors also noted that only a small percentage of patients with contrast extravasation detected by emergency contrast-enhanced CT were subsequently confirmed by angiography, thus, considered that the value of enhanced CT in predicting arterial injury was limited. The authors also believe that embolization of the main stem may cause incomplete hemostasis due to the abundant collateral circulations in the pelvic cavity. Although the author’s findings are mentioned in other studies, the article’s data and pictures only partially supported its inferences, and the conclusions cannot be drawn directly. In this Correspondence, we tried to reinterpret the additional findings in the article from our perspective. Through this discussion, we hope that more colleagues can re-understand the safety and effectiveness of non-selective internal iliac artery embolization in treating hemodynamically unstable pelvic fractures during the early resuscitation stage.

## Main text

We would like to comment on the article “High incidence of surgical site infection may be related to suboptimal case selection for non-selective arterial embolization during resuscitation of patients with pelvic fractures: a retrospective study” by Lai et al. [1], which was recently published in BMC musculoskeletal disorders.

The author pointed out that non-selective bilateral internal iliac artery embolization (nBIIAE) may lead to a higher rate of surgical site infection (SSI) for patients with pelvic fractures. However, the 11 patients with SSI mentioned included two patients with open pelvic fractures and six patients with Morel-Lavallée lesions. The authors indicated in the inclusion criteria that patients with open pelvic fractures were excluded. Additionally, open pelvic fractures themselves pose a higher risk of infection in the pelvic region [2]. Studies have also found a significantly increased risk of incisional infection in patients with Morel-Lavallée lesions who underwent pelvic fracture surgery [3]. It is debatable that the authors attribute the SSI after these two types of injury to nBIIAE.

The authors present a case of gluteus maximus necrosis, showing skin necrosis on both sides. Most of the right gluteus maximus was removed, and no muscle necrosis was confirmed on the left gluteus maximus. The embolic material on the right side of the patient was gelatin sponge. Considering the characteristics of the gelfoam, it cannot be excluded that the muscle necrosis was caused by the migration of gelfoam particles to the distal end of the artery. The trunk of the left internal iliac artery was embolized with a metal coil, and there was no evident necrosis on this side. This seems to have led to the opposite conclusion from the authors, which is that reliable trunk embolization does not lead to ischemia in the dominant region, whereas selective embolization may lead to ischemia or even necrosis of the organ [4, 5]. From the picture, the case is also considered a Moral-Lavallee injury. From the image during Internal iliac arteriography, the distal part of the bilateral internal iliac arteries can still be seen after embolization, indicating the presence of blood perfusion, which supports that the cause of the gluteus maximus necrosis was not due to embolization of the trunk of the internal iliac artery.

The authors also noted that only a small percentage of patients with contrast extravasation detected by emergency contrast-enhanced CT were subsequently confirmed by angiography, with the incidence decreasing from 82.9 to 26.3%. The authors considered that the value of enhanced CT in predicting arterial injury was limited. Some experts believe that the new generation of CT scanners is highly sensitive in detecting small bleeds, and these bleeding spots are potentially self-limited [6]. This may lead to significant differences between the results of CT and angiography. In addition, it is easy to understand that a ruptured artery may become occluded as a result of a decrease in arterial pressure and compression of the hematoma on the injured site. Also, hemostatic drugs are usually given early during treatment for hemodynamically unstable patients. These drugs can enhance the coagulation process or may promote vasoconstriction, making contrast extravasation not obvious. Furthermore, shock status and wire stimulation during the arteriographic process may result in arterial spasm, thus, lead to missed arterial injury [7]. Temporary cessation of bleeding due to these factors is not reliable for arterial hemostasis of pelvic fractures. Subsequent delayed bleeding may result in a shock state that is difficult to correct and may require re-intervention to stop the bleeding [6]. Non-selective internal iliac artery embolization may be the best choice at this time [5, 7]. In our experience, if a pelvic fracture is considered base on the injury mechanism and physical examination, a CT scan with three-dimensional reconstruction should be performed routinely. If there are no contraindications, choose contrast-enhanced CT as possible. For the trauma patients whose thoracic and abdominal organ injuries were excluded by FAST examination, considering that the bleeding is mainly caused by pelvic injury, the patients can be directly sent to the intervention room for internal iliac arteriography and embolization. The presence of contrast extravasation on pelvic CT scan is a strong indication for angiographic embolization, but negative results do not rule out arterial injury. We recommend using a steel ring to embolize the main trunk of the bilateral internal iliac arteries. Firstly, it can effectively prevent the displacement of the embolic material, block the blood supply to the pelvic cavity, and promote the hemostasis of the injured vessels. Secondly, as a means of damage control, nBIIAE can shorten the operation time as much as possible. Finally, nBIIAE can offer enough bleeding control by decreasing the blood flow of the internal iliac arteries and reduce the risk of rebleeding after resuscitation.

The authors suggest that embolization of the trunk may result in incomplete hemostasis due to the abundant collateral circulation in the pelvic cavity, which is supported by the presence of blood flow at the distal end of the internal iliac arteries during arteriography. However, this is inconsistent with the authors’ view that nBIIAE can lead to gluteus maximus necrosis. It was found that the pulse pressure decreased by 85%, and the blood flow decreased by 48% after ligation of both internal iliac arteries [8]. Bleeding in the injured area can be significantly reduced because of a significant decrease in pelvic blood flow perfusion, and the pulsatile bleeding of the artery can be significantly reduced because of the elimination of the trip-hammer effect of the artery. The blood clot is not easily dislodged, and venous-like hemorrhages occur in its place. The abundant collateral circulation in the pelvic cavity can ensure the blood supply of the organs [8]. In theory, the reduction of arterial blood flow can also help to reduce venous bleeding [7]. From our experience, bleeding from veins or bones can usually be controlled by pressure or packing, and blood clots can form with a strong chance of remaining in place after embolization [5, 9, 10]. In our previous studies [5, 10], a total of 22 patients with unstable pelvic fractures underwent nBIIAE for hemostasis, and only two died of hemorrhagic shock, with a success rate of 90.9%. No complications of pelvic ischemia were recorded, such as gluteus maximus necrosis, nonunion, lameness. Only one case of sexual dysfunction was reported. However, no evidence shows it is directly caused by nBIIAE. Therefore, we considered that nBIIAE might be an effective and safe way during the resuscitation stage in hemodynamically unstable pelvic fractures.

The management of hemodynamically unstable pelvic fractures remains a big challenge for trauma surgeons, and the effectiveness and applicability of various hemostasis measures are still controversial [11, 12]. The procedures for resuscitation should be based on the available resource and protocol of each trauma center. Performing nBIIAE on patients with specific injury types may lead to better outcomes.

## Data Availability

Not applicable.

## Abbreviations

nBIIAE: Non-selective bilateral internal iliac artery embolization
SSI: Surgical site infection
CT: Computed tomography

## References

[1] Lai C-Y, Tseng I-C, Su C-Y, Hsu Y-H, Chou Y-C, Chen H-W, Yu YH (2020). High incidence of surgical site infection may be related to suboptimal case selection for non-selective arterial embolization during resuscitation of patients with pelvic fractures: a retrospective study. BMC Musculoskelet Disord.

[2] Lunsjo K, Abu-Zidan FM (2006). Does colostomy prevent infection in open blunt pelvic fractures? A systematic review. J Trauma Inj Infect Crit Care.

[3] Suzuki T, Morgan SJ, Smith WR, Stahel PF, Gillani SA, Hak DJ. Postoperative surgical site infection following acetabular fracture fixation. Injury. 2010;41(4):396–9. 10.1016/j.injury.2009.11.005. 10.1016/j.injury.2009.11.005 20004894

[4] Cho J, Benjamin E, Inaba K, Lam L, Demetriades D. Severe bleeding in pelvic fractures: considerations in planning damage control. Am Surg. 2018;84(2):267–72. 10.1177/000313481808400236. 29580357

[5] Huang G-B, Hu P, Gao J-M, Lin X (2019). Analysis of early treatment of multiple injuries combined with severe pelvic fracture. Chinese J Traumatol.

[6] George CV. Pelvis. In: Ernest E. Moore, David V. Feliciano KLM, editor. Trauma. 8th ed. New York: McGraw-Hill Education; 2017. p. 677–91.

[7] Ramirez JI, Velmahos GC, Best CR, Chan LS, Demetriades D (2004). Male sexual function after bilateral internal iliac artery embolization for pelvic fracture. J Trauma.

[8] Burchell RC (1968). Physiology of internal iliac artery ligation. BJOG An Int J Obstet Gynaecol.

[9] Jin-Mou G, Xian-Yang T, Ping HU, Chang-Hua LI, Jian-Bai W, Jian-Bo Z (2005). Management of severe pelvic fracture associated with injuries of adjacent viscera. Chinese J Traumatol..

[10] Yang J, Gao JM, Hu P, Li CH, Zhao SH, Lin X (2008). Application of damage control orthopedics in 41 patients with severe multiple injuries. Chinese J Traumatol - English Ed.

[11] Duchesne J, Costantini TW, Khan M, Taub E, Rhee P, Morse B, Namias N, Schwarz A, Graves J, Kim DY, Howell E, Sperry J, Anto V, Winfield RD, Schreiber M, Behrens B, Martinez B, Raza S, Seamon M, Tatum D (2019). The effect of hemorrhage control adjuncts on outcome in severe pelvic fracture: a multi-institutional study. J Trauma Acute Care Surg.

[12] El Muntasar A, Toner E, Alkhazaaleh OA, Arumugam D, Shah N, Hajibandeh S (2018). Effect of angioembolisation versus surgical packing on mortality in traumatic pelvic haemorrhage: a systematic review and meta-analysis. World J Emerg Med.

